# Phase-modulating lasers toward on-chip integration

**DOI:** 10.1038/srep30138

**Published:** 2016-07-26

**Authors:** Yoshitaka Kurosaka, Kazuyoshi Hirose, Takahiro Sugiyama, Yu Takiguchi, Yoshiro Nomoto

**Affiliations:** 1 Central Research Laboratory, Hamamatsu Photonics K.K., Shizuoka 434-8601, Japan

## Abstract

Controlling laser-beam patterns is indispensable in modern technology, where lasers are typically combined with phase-modulating elements such as diffractive optical elements or spatial light modulators. However, the combination of separate elements is not only a challenge for on-chip miniaturisation but also hinders their integration permitting the switchable control of individual modules. Here, we demonstrate the operation of phase-modulating lasers that emit arbitrarily configurable beam patterns without requiring any optical elements or scanning devices. We introduce a phase-modulating resonator in a semiconductor laser, which allows the concurrent realisation of lasing and phase modulation. The fabricated devices are on-chip-sized, making them suitable for integration. We believe this work will provide a breakthrough in various laser applications such as switchable illumination patterns for bio-medical applications, structured illuminations, and even real three-dimensional or highly realistic displays, which cannot be realised with simple combinations of conventional devices or elements.

Several recent laser applications, such as maskless lithography^1^, super-resolution microscopy^2^, structured illumination^3^, and structured scattering^4^, involve elaborate beam-patterning control mechanisms. A high level of control is essential for generating desired mask patterns, doughnut-shaped beams, and stripe arrays. It has previously been achieved by transmitting or reflecting coherent light through phase-modulating elements, such as diffractive optical elements^5^ or spatial light modulators^5^. In these cases, because a light source with insufficient coherence produces a blurred pattern, gas or solid-state lasers have been used for their high level of coherence in other words high beam quality. However, their large component dimensions have hindered their miniaturisation and integration and obstructed the development of new applications utilising integrated devices. The integration of small on-chip device enables the development of new applications, encompassing both small- and large-scale integration. Small-scale applications include switchable illumination patterns for wearable modulated imaging^6^, switchable flow channels, or optical tweezers^7^ for biomedical applications (including flow cytophotometry^8^, tissue engineering^9^, and cell sorting^8^). Large-scale examples include real three-dimensional displays^10^ or highly realistic displays used in surgical settings, rendering glossy surfaces or realistic texture according to the viewing direction. Although semiconductor lasers are promising candidates for integration because of their compactness, high efficiency, and low power consumption (battery power is possible), achieving high beam qualities has been a significant challenge. However, semiconductor photonic-crystal surface-emitting lasers^11^,^12^,^13^,^14^,^15^ (PCSELs) have recently attracted much attention for their high beam quality^16^.

The PCSEL is a surface-emitting semiconductor laser that utilises a two-dimensional periodic structure (usually consisting of periodic air holes), namely a photonic crystal^17^, as a resonator. Lasing is realised via the two-dimensional distributed feedback effect^11^,^12^,^13^,^14^ of the photonic crystal. It is known that a photonic crystal significantly affects the characteristics of the light output from the PCSEL in terms of polarisation^18^, output power^16^,^19^,^20^,^21^, and beam pattern^22^,^23^,^24^. However, in PCSELs, the phase of the light cannot be configured arbitrarily, and therefore beam patterning has been demonstrated only in limited cases, such as for various types of doughnut-shaped^22^,^24^ and single-lobed^16^,^22^,^23^ beams, which have small divergence or are zero-dimensional patterns. In addition, these functions have been realised by changing the shape of the air holes or by introducing a phase shift^22^,^23^ as a defect line on a regular square or triangular lattice. Some ideas have recently been proposed for changing the beam direction where two lattices are combined^25^ or a periodic modulation is introduced^26^. However, the resulting beam patterns remain zero-dimensional.

We here introduce an internal holographic modulation of the lattice arrangement in a PCSEL to obtain freely configurable two-dimensional beam patterns. We employ a phase-modulating resonator in which the in-plane position of air holes is slightly shifted from a square lattice in a holographic manner. The arrangement is thus no longer periodic and the modulation must be imposed carefully so as not to compromise lasing. This novel on-chip-sized device, realising both lasing and phase modulation, is henceforth named an “integrable phase-modulating (iPM) laser”. We demonstrate that the iPM laser, which is small enough for integration, can emit characters, line drawings, multi-spot beams, and even grayscale images.

## Results

### Device structure

**Figure 1:**
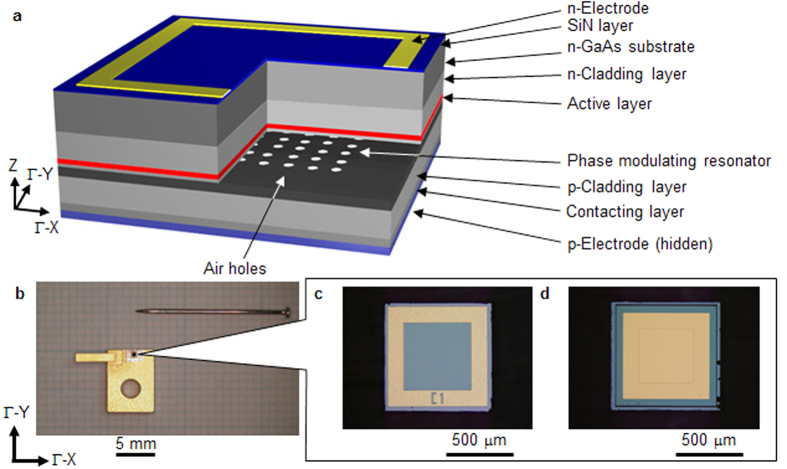
Device structure of the iPM laser. (**a**) Schematic device structure. (**b**) Top-view of the device, with the n-electrode side facing upward, placed on a sub-mount and an open heat sink. (**c**) Top-view of the device from the n-electrode side. (**d**) Top-view of the device from the p-electrode side.

Figure 1a shows the schematic device structure. The phase-modulating resonator and active layer are embedded between the cladding layers. Although this epitaxial structure is similar to that of the PCSEL^16^, the photonic-crystal layer in the latter, which utilises a two-dimensional periodic air-hole array, is replaced by a phase-modulating resonator as described below. Figure 1b is a photograph of the fabricated device mounted on an open heat sink and compared with the scale of a needle. Figure 1c,d are photographs of the device as seen from the perspectives of the n- and p-electrodes, respectively (see Methods - “Device structure”).

**Figure 2:**
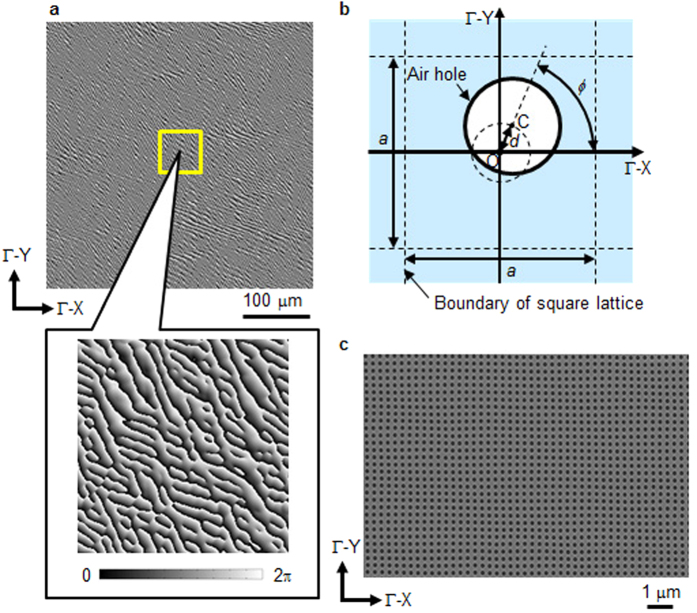
Phase-modulating resonator of the iPM laser. (**a**) Phase distribution of the phase-modulating resonator, consisting of 1400 × 1400 points. The grayscale indicates the phase. The lower picture shows an enlarged view of the area outlined by the yellow square. (**b**) Schematic of the phase-modulating resonator. The rotation angle *ϕ* corresponds to the phase distribution at each point in (**a**). (**c**) Top-view scanning-electron-microscope image of the phase-modulating resonator before becoming covered. This picture corresponds to a part of the 1400 × 1400 air-hole array.

**Figure 3:**
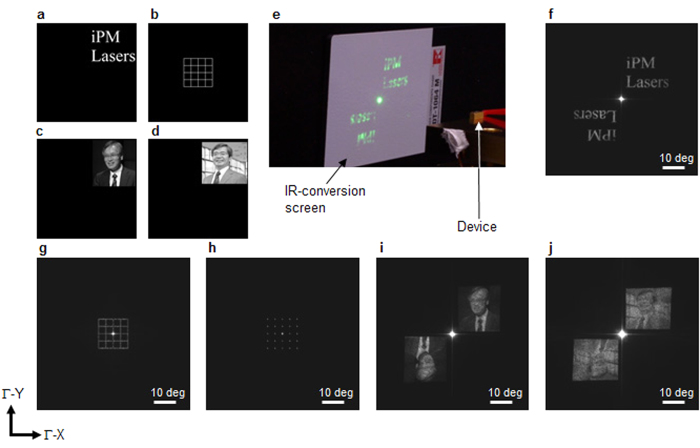
Far-field pattern of the iPM laser. (**a**–**d**) Target images. (**e**) Demonstration of lasing by the device with *d* = 0.04*a* using a driving current of 1.5 A under continuous-wave operation at room temperature (25 °C). The beam pattern was observed on an IR-conversion screen without any lens or scanning system. (**f**–**j**) Far-field patterns measured by a beam profiler ((**f**) characters spelling “iPM Lasers”; (**g**) grid pattern; (**h**) multi-spot beam; (**i**,**j**) photographic images). The detailed measurement conditions are described in the Methods section.

Figure 2 shows the top view of the phase-modulating resonator. This structure realises two important functions simultaneously: providing a standing-wave condition for lasing and phase modulation. A carefully designed small deviation from the square lattice enables sufficient two-dimensional feedback while retaining enough phase modulation. The phase distribution in Fig. 2a corresponds to the device that produces the beam pattern in Fig. 3f. Figure 2b shows a schematic view of the air-hole positions in the phase-modulating resonator. The dot labelled O indicates the lattice point of the square lattice, which is defined by the two axes labelled Γ-X and Γ-Y. The dashed grid indicates the unit cell, which has a lattice constant *a* = 282 nm (see Methods - “Device structure”). There is no air hole at the lattice point of the square lattice (point O). Instead, the centre (point C) of each air hole is on the perimeter of a circle of radius *d* = 0.06*a* or 0.10*a* (see Methods - “Device structure”) centred on O. The rotation angle *ϕ* is chosen according to the designed phase distribution, described in Methods - “Phase distribution of phase-modulating resonator”. This type of modulation is similar to the “detour-phase” method^27^ of the computer-generated hologram^5^. In addition, we introduced the following two ideas for designing the phase-modulating resonator, to retain the two-dimensional distributed feedback effect. Firstly, the air-hole size is kept constant in the phase-modulating resonator. During fabrication, the size of the air holes is not always proportional to that of holes before the growth of the following layer. In addition, air holes of different sizes are found at different depths to those achieved by dry etching. This disturbs the phase of the output beam, and therefore the air holes should be of the same size to achieve a precise control of the light wave. The second idea is that the distance between the lattice point (point O) and the centre of each air hole (point C) should also remain constant. If this condition is satisfied, when the phase is distributed uniformly from 0 to 2π over the phase-modulating resonator, the average central air-hole position approaches the lattice point of square lattice. In other words, the two-dimensional distributed feedback of the phase-modulating resonator approaches that of square-lattice photonic crystal. Figure 2c is a scanning electron microscope image of the phase-modulating resonator before the second growth step (see Methods - “Device structure”). The air holes have equal sizes and their positions are slightly shifted relative to the square lattice.

### Far-field patterns

We now focus on the main results. Figure 3 shows some far-field patterns produced by our iPM laser. Figures 3a–d show four target images. As shown in Fig. 3e, the iPM lasers emit these complicated beam patterns directly without requiring any lens or scanning system, and using only the power supply. No beam expander is necessary, only the iPM laser with an emission area of 400 μm × 400 μm. Figure 3f shows the far-field pattern that clearly displays the characters “iPM Lasers”, corresponding to Fig. 3a. The granular pattern might have been caused by fabrication error or speckle^28^. The twin mirror images might be due to the nature of the holography^5^ that accompanies the original images. The centre spot is likely to result from the modulation residue and may be suppressed (see Supplementary Information, Section 2). We also demonstrate that a grid-pattern (Fig. 3g) can be obtained as well as multi-spot beams (Fig. 3h). The former result indicates that an arbitrary flow channel can be created and switched by integration. Encouraged by these results, we then tried to generate more generalised and complicated patterns: grayscale images. Figure 3i,j show emitted grayscale images corresponding to the black and white backgrounds in Fig. 3c,d, respectively. Like computer-generated holograms, these complicated images consist of many spots. Here, the image with the white background has many spots, but both portraits are nevertheless clearly recognisable despite the granularity introduced by fabrication error or speckle. These few examples suffice to illustrate the versatility of iPM lasers for generating arbitrary patterns.

## Discussion

**Figure 4:**
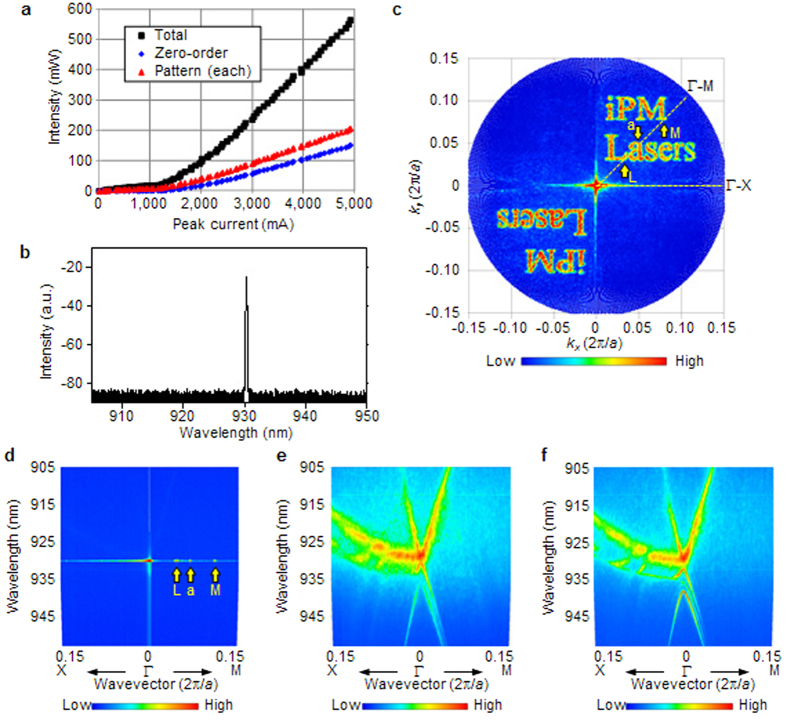
Angular and wavelength dependences of the device. (**a**) Output power versus current for the device corresponding to Fig. 3f. (**b**) Lasing spectrum corresponding to Fig. 3f. (**c**) Angular dependence of the device at the lasing wavelength. (**d**,**e**) Angular and wavelength dependences of the device sliced along the Γ-X and Γ-M directions indicated by dashed lines in (**c**) above (4 A) and below (400 mA) the lasing threshold current, respectively. (**f**) Angular and wavelength dependences of a square-lattice PCSEL below (400 mA) the lasing threshold current. The detailed measurement conditions are described in the Methods section.

As demonstrated above, iPM lasers can successfully emit arbitrarily configured light patterns, implying that phase modulation is fully achieved in our devices. We now turn to the lasing mechanism. Figure 4a shows the output power versus current characteristics of the device used in Fig. 3f. “Total” and “Zero-order” denote the powers of the total output and centre spot, respectively. The output power of “Pattern (each)” is calculated from the measured values of “Total” and “Zero-order” (see Methods - “Measurement of output power versus current”). Figure 4b shows the lasing spectrum of the device used in Fig. 3f, under the same driving condition (see Methods - “Measurement of lasing spectra”). The lasing spectra for the other devices are also shown in Supplementary Fig. S3. Only one peak is observed in each case, indicating the formation of a single standing wave. In the PCSELs, more information about the in-plane standing wave can be obtained by measuring the angular and wavelength dependences^29^,^30^ of the device. It is called a band structure^17^ and the standing-wave condition corresponds to a band edge^17^ (see Supplementary Information, Section 3). We measured both the angular and wavelength dependences of the device used in Fig. 3f. Figure 4c shows the angular dependence at the lasing wavelength (see Methods - “Measurement of angular distribution of emission spectra” for measurement details). The same pattern is observed as in the measured far-field pattern in Fig. 3f, indicating that the far-field pattern consists of one wavelength. Figure 4d,e show slices taken along the dashed lines in Fig. 4c above (4 A) and below (400 mA) the lasing threshold current (~1.3 A), respectively. In Fig. 4d several peaks exist along the Γ-M direction, indicated by arrows. These correspond to points ‘L’, ‘a’, and ‘M’ in Fig. 4c. However, the peaks are absent in Fig. 4e, implying that the far-field pattern is observed only above the threshold current and that there is no resonance structure or band edge corresponding to the points ‘L’, ‘a’, and ‘M’. Here, the absence of an applied perturbation to the phase-modulating resonator of an iPM laser corresponds to a square-lattice PCSEL. The lasing mechanism of iPM lasers should therefore be similar to that of a square-lattice PCSEL for sufficiently small perturbations. Figure 4f shows the angular and wavelength dependences of a square-lattice PCSEL with the same air holes in the absence of any perturbation. This corresponds well with Fig. 4e. These observations imply that the two-dimensional standing wave for lasing is formed in a way similar to square-lattice PCSELs (see Supplementary Information). In conventional PCSELs, part of the in-plane light wave is scattered with the same phase across the photonic crystal, leading to a plane wave propagating in the normal direction and giving rise to a single-spot beam. In contrast, in iPM lasers, the light wave is diffracted at a point that deviates from the square lattice in-plane, as determined by the designed radius *d* and rotation angle *ϕ*. Therefore, the diffracted light wave propagating in the surface-normal direction has a phase that deviates locally at each point, eventually forming a phase distribution. As a result, a finely modulated wave front is emitted and arbitrary beam patterns can be obtained. As the resonance mechanism is similar to that of PCSELs, the operating wavelength of iPM lasers can be scaled^17^. In other words, by changing the designed lattice constant *a* and by choosing an appropriate active medium, the emission wavelength can be controlled. Furthermore, devices for different wavelengths can be integrated into a single module.

In conclusion, we have proposed a class of new devices referred to as “iPM lasers”, which comprise a phase-modulating light source, suitable for on-chip integration. These devices involve a structure with concurrent phase-modulation and lasing functionality. We demonstrated the operation of semiconductor iPM lasers that emit characters, line drawings, multi-spot beams, and even grayscale images. These results suggest that iPM lasers can emit arbitrary beam patterns. We also discussed the working mechanism of the devices. We believe that iPM lasers will open a new field of applications which ranging from the small scale integration such as switchable illumination patterns for wearable modulated imaging^6^, switchable flow channels, or optical tweezers^7^ for biomedical applications (including flow cytophotometry^8^, tissue engineering^9^, and cell sorting^8^), to the large scale integration such as real three-dimensional displays^10^ or highly realistic medical-imaging displays that render glossy surfaces or realistic textures.

## Methods

### Device structure

Figure 1a shows the fabricated device structure. The total chip size is 800 μm × 800 μm. An n-cladding layer, active layer, phase-modulating resonator, p-cladding layer, and contacting layer were epitaxially grown onto an n-GaAs substrate. This process is divided into three steps. First, the n-cladding layer, active layer, and phase-modulating resonator were grown on the substrate. A 1400 × 1400 hole array was then fabricated on the phase-modulating resonator by electron-beam lithography (JEOL, JBX-6300SP) and dry etching (SAMCO, RIE200iP). The patterned area of the phase-modulating resonator covers 400 μm × 400 μm. Next, the p-cladding layer and contacting layer were epitaxially grown on the patterned phase-modulating resonator. During growth, air holes were formed at the etching holes, owing to the difference in growth rate between the interior and exterior of the etching holes. Finally, an n-electrode, with a window-like opening above the emission area, was deposited onto the n-GaAs substrate and a SiN anti-reflection layer was coated onto the opening. A p-electrode with 400 μm × 400 μm contact area was also deposited onto the contacting layer. Injected electrons from the n-electrode and injected holes from the p-electrode interact in the active layer and radiate upon recombination. The radiated light wave undergoes two-dimensional diffraction within the plane and a standing wave is formed by diffraction at the air holes (see Supplementary Information for details). Lasing occurs when sufficient current is applied. We note that the distance between the n-electrode and the active layer exceeds 100 μm, whereas that between the p-electrode and the active layer is only a few microns. Therefore, the lasing area is almost equal to the contact area at the p-electrode. The lattice constant *a* was 282 nm for the devices used in Figs 3f–j and 4, and 284 nm for that in Fig. 3e. The threshold current depends on the target images and also on the radius *d*. The smaller *d*, the lower the threshold current. On the other hand, large values of *d* diminish the zero-order light (see Supplementary Information, Section 2). We designed *d* by taking into account the above features, setting *d* = 0.04*a* for the device in Fig. 3e (measured by direct-current power supply, THORLABS, LDC220), 0.06*a* for those in Fig. 3f,i,j, and 0.10*a* for those in Fig. 3g,h. The devices were mounted onto an open heat sink.

### Measurement of far-field patterns

The far-field patterns were measured using a far-field optical system (HAMAMATSU, A3267-12) and a beam profiler (HAMAMATSU, LEPAS-12). Each image was acquired by averaging 8 times. The measurements were carried out under pulsed operation to suppress thermal effects at room temperature (25 °C). We used a pulsed current source (ILX Lightwave, LDP-3830) and a current probe (YOKOGAWA, 701933). The pulse width and frequency were 50 ns and 10 kHz, respectively. The driving current was 4 A. To enhance visibility, the central zero-order spot was saturated.

### Measurement of output power versus current

The output power was measured using a photodiode (OPHIR, PD300-3W-V1) and measuring software (OPHIR, Starlab) with no ND filter. Starlab parameter settings for the wavelength, range, and averaging interval were 930 nm, “auto”, and 1 s, respectively. The measurements were carried out under pulsed operation at room temperature (25 °C). We used a pulsed current source (ILX Lightwave, LDP-3830) and a current probe (YOKOGAWA, 701933). The pulse width and frequency were 50 ns and 10 kHz, respectively. The distance between the device and the photodiode was approximately 3 mm for “Total” and 150 mm for “Zero-order”, respectively. The output power of “Pattern (each)” was calculated as


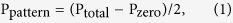


where P_total_ and P_zero_ are the measured output powers for “Total” and “Zero-order”, respectively. We calculated P_pattern_ because the size of the beam pattern at a distance of 150 mm exceeds the measurement area of the photodiode and cannot be measured directly.

### Measurement of lasing spectra

The lasing spectra were measured using an optical spectrum analyser (YOKOGAWA, AQ6373) through a single-mode fibre. The resolution, sensitivity, and averaging time were 0.02 nm, “High 2”, and 1, respectively. The measurements were carried out under pulsed operation at room temperature (25 °C). We used a pulsed current source (ILX Lightwave, LDP-3830) and a current probe (YOKOGAWA, 701933). The pulse width and frequency were 50 ns and 10 kHz, respectively. The driving current was 4 A.

### Measurement of the emission-spectra angular distribution

The measurement setup consisted of a multi-channel spectrometer (HORIBA, iHR320), multi-mode optical fibre, lens, and rotating arm (see Supplementary Fig. S1). Emission from a device in a specific direction was measured using a multi-channel spectrometer through a multi-mode optical fibre. We defined spherical polar coordinates around the device. The polar angle *θ*_tilt_ and the azimuthal angle *θ*_rot_ were transformed into the normalised wavevector (*k*_*x*_, *k*_*y*_) in reciprocal space as follows:


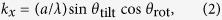



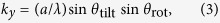


where *k*_*x*_ and *k*_*y*_ are the wavevectors parallel to the Γ-X and Γ-Y directions, *a* is the lattice constant, and λ is the wavelength in air. In Fig. 4c, the horizontal axis corresponds to *k*_*x*_ and the vertical axis corresponds to *k*_*y*_. In Fig. 4d–f the horizontal axis indicates the wavevector, which is *k*_*x*_ in the Γ-X direction and 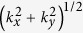 in the Γ-M direction. The vertical axis corresponds to λ and the colour scale indicates the light-wave intensity as a function of the wavevector and wavelength. The measurements were carried out under pulsed operation at room temperature (25 °C). We used a pulsed current source (ILX Lightwave, LDP-3830) and a current probe (YOKOGAWA, 701933). The pulse width and frequency were 50 ns and 10 kHz, respectively. The driving currents were 4 A, 4 A, 400 mA, and 400 mA for Fig. 4c–f, respectively.

To obtain the angular dependence of the device at the lasing wavelength (Fig. 4c), the measurements taken along each direction were stored in the 3D arrays for (*k*_*x*_, *k*_*y*_, λ). Then, a horizontal cross section through this array at a desired wavelength yields the angular dependence of the device. The colour scale indicates the light intensity.

### Phase distribution of the phase-modulating resonator

We refer to the method of computer-generated holograms^5^ to obtain the phase distribution of the phase-modulating resonator. The target beam pattern is related to the complex field distribution on the phase-modulating resonator via Fraunhofer diffraction, namely a Fourier transform^5^. In other words, the complex field distribution on the phase-modulating resonator can be obtained by performing the inverse Fourier transform on the target beam pattern. In concrete terms, we prepared a bitmap image of the target beam pattern. Then, using the fast Fourier transform algorithm, the complex field distribution on the device (the inverse Fourier transform of the beam pattern) was obtained using the “ifft2” function in analysing software (Mathworks, MATLAB). Then, we extracted the phase distribution of the phase-modulating resonator from the complex field distribution on the device using the “angle” function in the software.

## Additional Information

**How to cite this article**: Kurosaka, Y. *et al.* Phase-modulating lasers toward on-chip integration. *Sci. Rep.* **6**, 30138; doi: 10.1038/srep30138 (2016).

## Supplementary Material

Supplementary Information
