# Supplementary material for: Phase-modulating lasers toward on-chip integration
Source: Sci Rep. 2016 Jul 26;6:30138. doi: 10.1038/srep30138 (PMC4960481; doi:10.1038/srep30138)
Supplement: Supplementary Information [file srep30138-s1.pdf]

Supplementary information

Phase-modulating lasers toward on-chip integration

Yoshitaka Kurosaka*, Kazuyoshi Hirose, Takahiro Sugiyama, Yu Takiguchi and Yoshiro Nomoto

Central Research Laboratory, Hamamatsu Photonics K.K., Shizuoka 434-8601, Japan.

***e-mail: yoshitaka.kurosaka@crl.hpk.co.jp

Supplementary methods

1. Resonance and wavefront modulation mechanism of iPM lasers with and without perturbation of air-hole positions

1-1. Without perturbation (square-lattice PCSEL)

In the absence of an applied perturbation, the phase-modulating resonator of an iPM laser (d = 0) corresponds to a square-lattice PCSEL. This subsection discusses the resonance mechanism in square-lattice PCSELs. Supplementary Fig. S2a indicates the four main light waves that are present under the resonance condition in reciprocal

space.[16] A lattice constant a in real space gives a reciprocal lattice vector $2\pi/a$ in reciprocal space. The black dots indicate the reciprocal lattice points and the black arrows the wavevectors of the main light waves. The short red arrows indicate the fundamental lattice vector with magnitude $2\pi/a$. The wavevector magnitudes of the four main light waves is $2\pi n/\lambda$, which equals $2\pi/a$, where λ is the wavelength in air and n the effective refractive index. Therefore, the magnitude of the four main lightwaves corresponds to that of the fundamental reciprocal lattice vector. The diffraction effect can be described as a vector addition of the reciprocal lattice vectors and of the main light waves. For example, the reciprocal lattice vector associated with diffraction in the reverse direction corresponds to twice the fundamental reciprocal lattice vector in the reverse direction. Overall, these four main light waves are mutually coupled via diffraction in the lattice. They are also diffracted towards the normal direction by diffraction of the fundamental reciprocal lattice vector, maintaining their original magnitude (see Supplementary Fig. S2b). More details are given in Ref. 16.

1-2. With perturbation (iPM lasers)

In the case of iPM lasers, the air-hole positions do not coincide with the square lattice ($d \neq 0$), as indicated in Fig. 2b. Since the air holes are centred (points C) on the perimeter

of a circle centred on point O in Fig. 2b, and these positions vary according to the phase distribution, the distance between neighbouring air holes also varies. However, as shown in Fig. 4e, the observed angular and wavelength dependences correspond to that of the square-lattice structure (Fig. 4f), and lasing occurs at the band edge of the square-lattice structure (Figs. 4d and e). We can therefore conclude that the perturbation is sufficiently small that lasing in the square-lattice structure is not prevented. In other words, light at the lasing wavelength forms a two-dimensional standing wave in the in-plane directions (Γ-X and Γ-Y directions). The light wave is then diffracted in the vertical direction (Z direction). The diffraction process can be understood in the following two steps. Firstly, scattering occurs at a boundary between media with different refractive indices, in our case at the air-hole boundaries. Next, the scattered light waves interfere with each other. For the square-lattice structure, the air holes are arranged periodically and the scattered light waves form a plane wave in the vertical direction. In contrast, the air holes in iPM lasers are not periodic, and therefore the scattered light waves deviate from a plane wave, so that the phase is modulated according to the rotation angle ϕ for each air hole.

2. Suppression of zero-order light

As mentioned in the main text, the central spot (referred to as zero-order light) is thought to arise from a modulation residue. It is therefore expected to decrease by increasing the degree of modulation. This can be achieved by increasing *d* in Fig. 2b. In practice, both the zero-order light and the twin image can easily be removed by placing a shutter above the device. In addition, the suppression of zero-order light and twin images in a computer-generated hologram is discussed in reference S1. However, our goal is to demonstrate the generation of arbitrary beam patterns, and therefore the suppression of zero-order light is outside the scope of the present article. This issue will be discussed in a further publication.

3. Band edge of the photonic band structure

In photonic crystals, including square-lattice PCSELs, the angular and wavelength dependences relate to the photonic band structure.[17] In this structure, the zero-gradient point is known as the band edge. The light-wave group velocity at the band edge is zero. Lasing oscillation is considered to occur at the band edges of the Γ point where the feedback effect reaches maximum intensity.[12] In iPM lasers, lasing takes place at the band edge of the Γ point, as shown in Figs. 4d and e. This indicates that the formation

of a two-dimensional standing wave for lasing occurs by means of the two-dimensional distributed feedback effect in iPM lasers, as also occurs in square-lattice PCSELs.

References

S1. Toal, V. *Introduction to holography, Ch.13.4. Suppression of the zero-order and the twin image* (CRC Press, Boca Raton, 2012).

Supplementary Figures and Legends

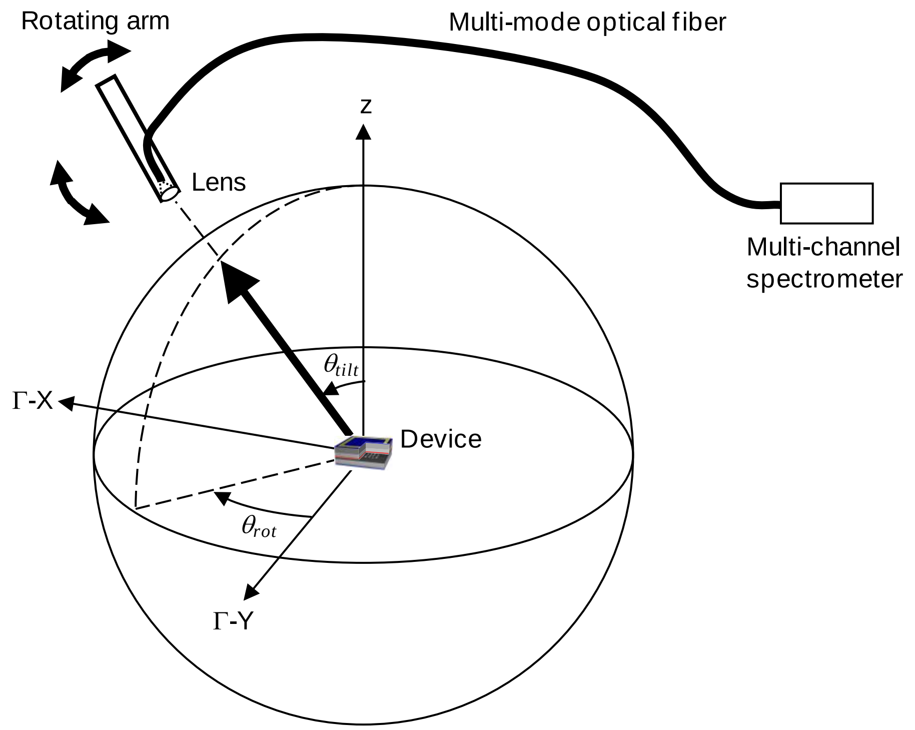

Supplementary Figure S1 | Setup for measuring the angular and wavelength dependences of the device.

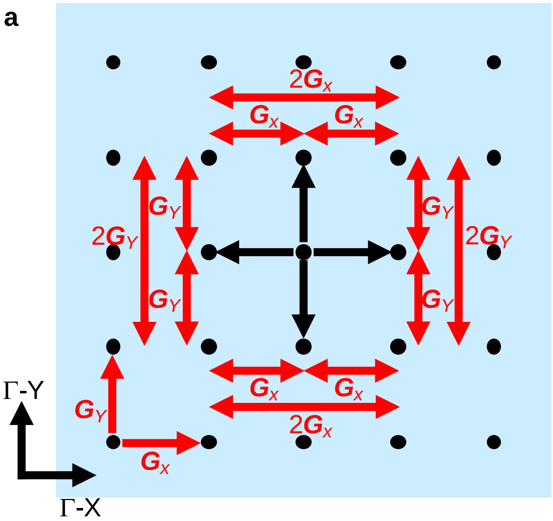

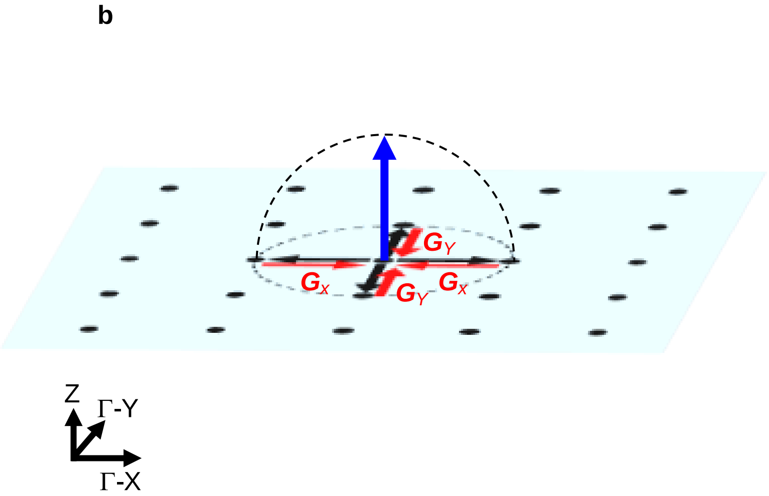

Supplementary Figure S2 | Light wave coupling in reciprocal space. (a) In-plane coupling. (b) Coupling to the vertical direction from the in-plane directions.

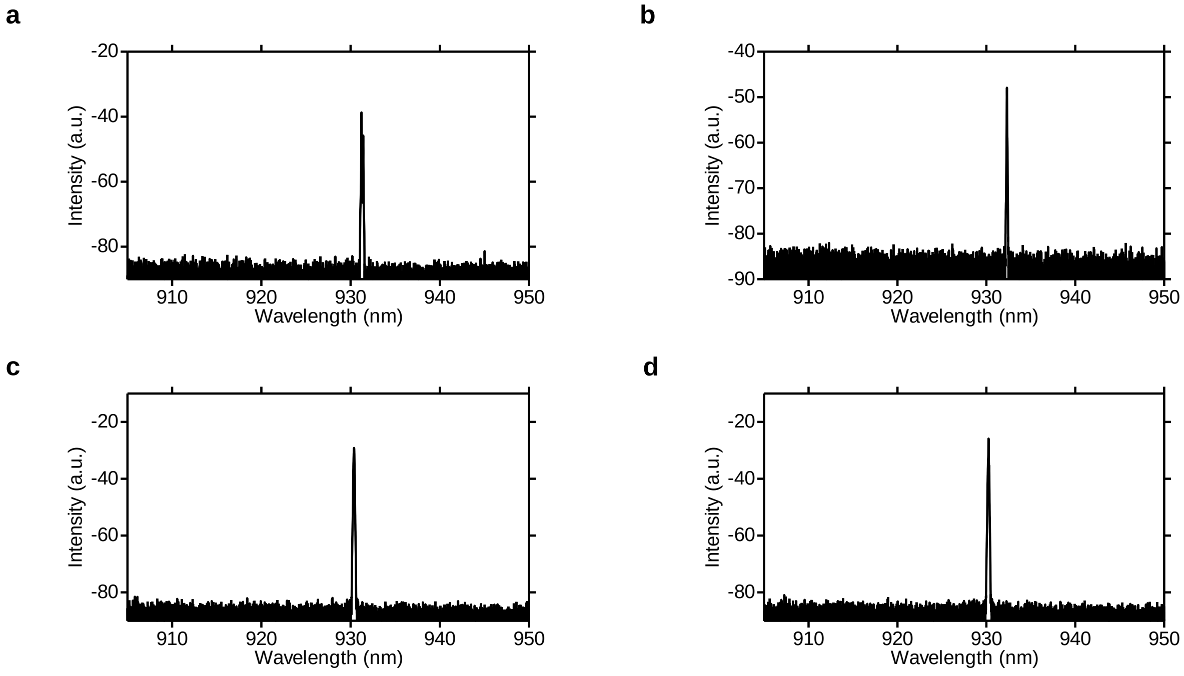

Supplementary Figure S3 | Lasing spectra. The lasing spectra shown correspond, respectively to (a) Fig. 3g, (b) Fig. 3h, (c) Fig. 3i, and (d) Fig. 3j.
